# ‘Care Is Lost When You Get Jumbled Around’—Aboriginal Grandmothers and Family Perspectives of Cultural Continuity in Healthcare as Recipients of Care in Mainstream Health Services in South Australia

**DOI:** 10.1007/s40615-025-02564-w

**Published:** 2025-07-24

**Authors:** Nina Sivertsen, Tahlia Johnson, Janiene Deverix, Susan Smith, Julian Grant

**Affiliations:** 1 College of Nursing and Health Sciences, Caring Futures Institute, Flinders University, Adelaide, SA Australia; 2 Faculty of Health Sciences, UiT Arctic University of Norway, Tromsø, Norway; 3 Manager Aboriginal Services, Child and Family Health Service, Women’s Children’s Health Network, 295 South Terrace, Adelaide, SA 5000 Australia; 4 Women’s and Children’s Division, Flinders Medical Centre, Southern Adelaide Local Health Network, Adelaide, SA Australia; 5 School of Nursing, Midwifery, Charles Sturt University, Panorama Ave, Bathurst, NSW 2795 Australia

**Keywords:** Continuity of care, Access and barriers to healthcare, Culture in care, Aboriginal health, Community care

## Abstract

Aboriginal women and their infants face substantial health disparities compared to non-Aboriginal women and their infants. Timely, effective, and culturally appropriate maternal and child healthcare can address these inequalities. However, many Aboriginal women experience fear and anxiety when using mainstream healthcare services, leading to lower attendance at perinatal appointments, often due to inadequate communication, poor service coordination, and a lack of continuity in care. This research sought to explore factors that contribute to continuity of care and to consider service features that contribute to positive care experiences and satisfaction with care received by Aboriginal women and their infants. This qualitative study used yarning to explore the experiences and perceptions of care of nine Aboriginal families. Three main themes were identified: (1) Bringing culture to the centre of healthcare with subthemes; (2) Care is lost when you get jumbled around, and (3) In and out for check-ups, scans, and things—no one asked if I needed help. The findings of this research highlight a lack of continuity of care for Aboriginal families accessing mainstream health care services in South Australia, from the antenatal period through to an infants’ first 2000 days of life. This research identified strategies for enhancing continuity, enabling communities and healthcare services to provide appropriate and culturally safe care. By implementing culturally safe and appropriate care, health disparities can be reduced, maternal and child health outcomes may improve, and trust can be fostered between Aboriginal communities and mainstream healthcare services.

## Introduction

The first 2000 days of life, from conception through to a child’s second birthday, are critical for long-term health and well-being. For Aboriginal families, this period is particularly important, as health disparities and barriers to accessing culturally safe care can significantly impact maternal and infant health outcomes [1]. These disparities are clear in maternal and infant health outcomes with Aboriginal women experiencing higher rates of gestational diabetes and smoking during pregnancy. Rates of preterm birth and low birth weight are nearly double among Aboriginal infants, perinatal mortality rates of Aboriginal infants are 50% higher than those of non-Aboriginal infants, and maternal mortality rates of Aboriginal women nearly three times higher compared to their non-Aboriginal counterparts [1–3]. Aboriginal women are less likely to attend mainstream health services, commence antenatal care at the recommended time, and attend the recommended number of antenatal visits than non-Aboriginal women [1, 2].

Compared to non-Indigenous Australians, Aboriginal populations experience a significant level of disadvantage in health, life expectancy, education, employment, and living standards [4]. Maternal and infant health worsens with increasing remoteness, due to challenges in health service provision and delivery [3]. This disproportionally affects Aboriginal women and their infants, as 26% of Aboriginal births occur in remote or very remote areas, compared to only 2% of non-Aboriginal births [2, 3]. However, healthcare services can contribute to a reduction in health disparities between non-Aboriginal women and Aboriginal women and their infants, through the provision of timely, effective, and culturally safe maternal and child healthcare [2].

Continuity of care, where healthcare is coordinated and provided by consistent and culturally aware practitioners, has been reported as being more culturally safe than siloed care and can result in greater uptake in health care during the perinatal period [20]. As health disparities continue to exist for Aboriginal women and infants, it is essential to explore the factors that contribute to this. Continuity of care is considered central to the provision of culturally safe care; however, little is known about how Aboriginal families experience continuity of care during this crucial time [1, 5]. A systematic literature review by Sivertsen et al. [1] analysed 62 studies to assess whether they explicitly addressed continuity of care. The study found that a lack of continuity of care, increased medical risks, and compromised safety, leading to adverse outcomes for Aboriginal women and infants, and poor engagement with services due to lack of culturally safe experiences. However, embedded Aboriginal services in midwifery, was shown to be well accepted by Aboriginal families.

This study explores the experiences of Aboriginal families in accessing and receiving continuous care for the family throughout the first 2000 days of their infants’ life in South Australia. The first 2000 days of framework is a research-based approach aimed at supporting early childhood development from conception to nearly 5 years old, focusing on promoting optimal physical, mental, and social-emotional growth during this crucial period [6]. By understanding the perspectives of Aboriginal families, this research identifies factors that either support or hinder continuity of care, with the goal of improving service delivery and ensuring that care is culturally safe, responsive, and beneficial to Aboriginal communities. The findings from this study will contribute to a deeper understanding of how healthcare systems can better meet the needs of Aboriginal families during this vital period.

## Method

The aim of this study was to (1) explore and identify health workers’ capacity to provide culturally safe continuity of care to Aboriginal families with infants in the first 2000 days of life and (2) explore with Aboriginal families their experiences of care as continuous during the first 2000 days of their infants’ life. Health care providers’ perspectives can be found in Sivertsen et al. [7]. This paper shares the voices of Aboriginal families who yarned with the research team about their perceptions and experiences of care provision.

Yarning as a research method is deeply embedded in Indigenous methodologies, particularly within Aboriginal and Torres Strait Islander cultures in Australia [8]. Yarning is the most frequently used Aboriginal research method in Aboriginal qualitative health research in Australia. This method privileges Aboriginal knowledge systems through interrelatedness and relationships [8]. The Yarning method is a valid research technique for decolonising research practice [8], if undertaken sensitively with Aboriginal researchers. This method transcends conventional research techniques by prioritising storytelling, personal experiences, and mutual respect, offering a culturally congruent approach to engaging with Aboriginal communities [8]. At its core, the goal of yarning is to establish a conversational environment where participants and researchers can share knowledge and stories in a safe space. Unlike structured interviews or surveys, yarning allows for a fluid, dynamic interaction that can adapt to the needs and comfort levels of the participants. This flexibility makes it an invaluable tool in Aboriginal research, where respecting cultural protocols and building genuine relationships are paramount [8, 9].

### Eligibility Criteria

This qualitative study was conducted in collaboration with a state-wide mainstream health service responsible for providing care to Aboriginal families across the first 2000 days in Adelaide, South Australia. Aboriginal families receiving care by practitioners working as Aboriginal Cultural Child and Family Support Consultants (ACCFSCs), Aboriginal Maternal Infant Care (AMIC) workers, midwives or Child and Family Health Nurses (CaFHNs) (Child and Family Health Nurses) in seven metropolitan service areas and one rural service area, were invited to participate in the study. All Aboriginal and Torres Strait Islander families using these services were eligible to participate in this study.

### Recruitment

An Aboriginal community researcher recruited participating families through study sites in clinical and community settings and took the time to establish a personal relationship with each family. This approach fostered trust and ensured that participants felt comfortable and supported throughout the study. The recruitment process included a combination of convenience and purposive sampling. Recruitment began with a convenience sample to quickly engage participants and establish initial contacts and build trust within the community. Purposive sampling techniques were then applied to intentionally select participants from diverse backgrounds and experiences, ensuring that the study captured a comprehensive range of Aboriginal family perspectives relevant to the research objectives. All participants provided informed consent prior to their involvement, with the consent process including a thorough explanation of the study’s purpose, procedures, and potential impacts. Consent was obtained in a culturally sensitive manner, adapting the process to respect and align with the cultural values, communication styles, and social practices of the participants while allowing participants ample opportunity to ask questions, consult with family, and withdraw at any stage if they wished. In this study, obtaining consent in a culturally sensitive manner which included building trust by taking time to explain the study’s purpose and procedures clearly and addressing any potential concerns in an approachable, respectful way. Communication was kept accessible, avoiding overly technical language. Additionally, Aboriginal cultural protocols, such as consulting with Elders or community leaders, were observed to honour community practices. This approach fostered respect and inclusivity, allowing participants to feel informed, comfortable, and autonomous in their decision to provide consent. Participants were compensated for their time and expenses in the form of a voucher.

The participants are service users of a large portfolio of health networks providing services to people in South Australia [10]. The Aboriginal Family Birthing Program (Ngangkita Ngartu) is the Kaurna name for this service, which means caring for Aboriginal women during pregnancy, a program based on Aboriginal Culture Grandmothers Law) postnatal care in a culturally sensitive environment, with the support of midwives, doctors, AMIC workers, social workers, and family support workers [10].

### Yarning Sessions

The yarning sessions were facilitated by an Aboriginal community researcher. Yarning sessions were conducted with nine families comprising 19 participant carers of 25 children 0–5 years of age: mums (*n* = 7), grandmothers (*n* = 7), aunties (*n* = 4), and one dad (*n* = 1). Not all members of each participant family were available to yarn with the team (see Table 1 for participants). Participant ages ranged from 23 to 72, and the children in each family ranged from 1 to 8. Services engaged with included GP; midwives; Aboriginal health services; CAFHS (Child and Family Health Service); and community organisations and clinics. Participants were provided with an information sheet, and written consent was obtained prior to the yarning (see Table 1—Participant families). During the yarning sessions, which lasted from 45 min to 1 h and 30 min, participants were asked about their experience of accessing health services, continuity of care, management of transitions between services, and cultural safety.

**Table 1:**
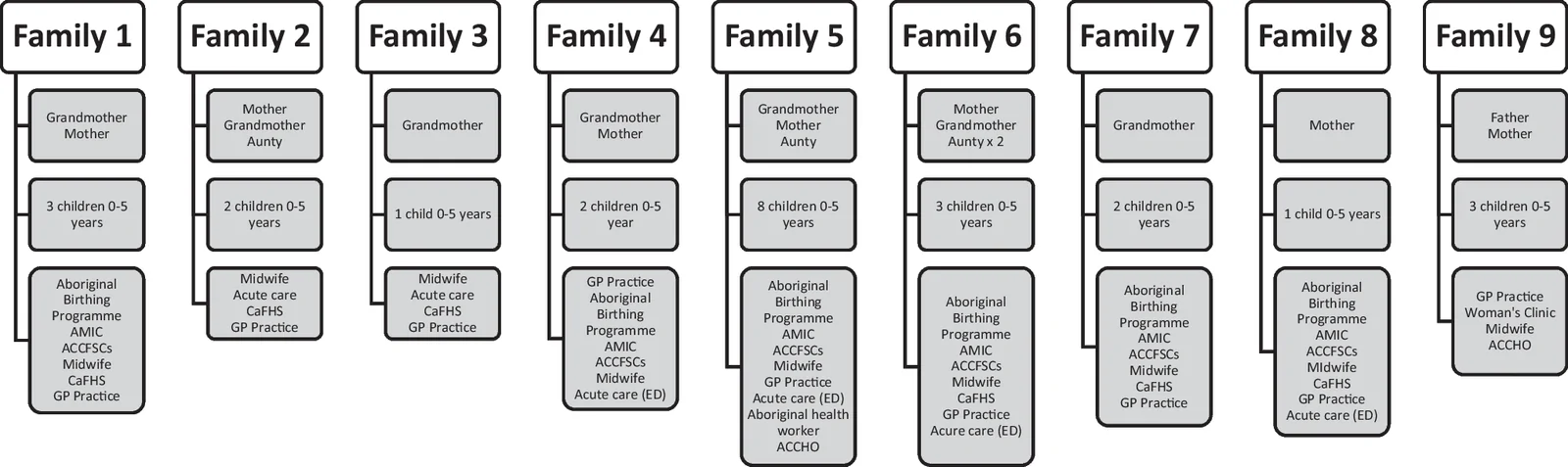
Participant families

### Analysis

The yarning was audio recorded, transcribed, and uploaded into NVivo 12 (then updated in NVivo 14 [11], and four joint and collaborative sessions were undertaken to yarn together about the data as a research group, comprising both Aboriginal and non-Aboriginal researchers. Data were analysed using thematic analysis [18]. The collaborative sessions ensured that rigor and quality were maintained by using two-eyed seeing in the data analysis [19].

## Results

Three main themes were identified in this study including: (1) bringing culture to the centre of healthcare; with sub themes equity in service set up—online not suitable for all: (1b) healthcare workers lack of cultural awareness, (1c) what worked well, (1d) Aboriginal roles supporting Aboriginal families, and (1e) increased awareness of Aboriginal cultures and understood families’ health needs; (2) care is lost when you get jumbled around with sub themes: (2a) a familiar face, (2b) judgement, (2c) systems within systems, and (2d) you lose care being jumbled around; and (3) in and out for check-ups, scans, and things—no one asked if I needed help.

### Bringing Culture to the Centre of Health Care

The first key theme emphasises the importance of integrating cultural understanding into mainstream healthcare services. This theme explores two critical aspects: identifying what needs to change in current healthcare practices and recognising what has worked well for families accessing these services.

But I guess she probably felt more alone with that situation, where if she was trying to voice her concern for something or voice her opinion or… Or voice something, she might not have been understood as well, as I guess a typical white person would be…most of the midwives and nurses were all white, and people that did our scans were white, people that run the courses were white. Different world really….if you have a little bit of trouble with, yeah, the English language or anything like that, it could be a bit isolating I guess (Family 9)

### Equity in Service Set up—Online Not Suitable for All

Several families (*n* = 5) expressed concerns about the lack of equity in the setup of health services, particularly when accessing care through online platforms. Many Aboriginal families, especially those living outside of metropolitan areas, may have limited or unreliable internet access or lack digital literacy, making it difficult to use these services or may simply prefer face-to-face interactions, particularly when discussing sensitive health issues.

You’ve got a lot of programmes online…But a lot of parents who I find, like where we were, Aboriginal parents, a lot of them don’t come from internet. So therefore, they can’t do the research. A lot of them can’t read…So therefore they can’t learn, they need someone physically to see them (Family 3).

Cultural barriers play a significant role. For many Aboriginal families, the personal connection and trust that come from in-person interactions are essential to feeling understood and respected in a healthcare setting.

People need to be genuine in wanting to help… Because Aboriginal people are very sensitive and know when people don’t really give a damn about them and when they are being judgemental. … [healthcare workers] think they aren’t judgemental when they actually even are (Family 7)

The shift toward online services can strip away these culturally important aspects of care, further marginalising those who already face barriers to accessing mainstream healthcare. As a result, the reliance on digital platforms risks creating a two-tiered system where those who cannot or do not wish to engage with online services are left behind, exacerbating existing healthcare inequities.

Before you have a child, they don’t sort of go, okay, here’s your list, check these off. Go to these people, see this sort of thing. It’s not, I guess set out for new parents, so if you don’t know, which a lot of new parents wouldn’t, you don’t seek a service, you don’t think about it until after when you realise, oh yeah, hang on, we could’ve done that before this happened, and all those sorts of things (Family 9).

### Health Care Workers’ Lack of Cultural Awareness

All nine families (*n* = 9) in the study expressed a common concern that healthcare workers lacked cultural awareness and the skills necessary to provide culturally safe care. They expressed feelings of being dismissed and not heard by healthcare providers, which led to feelings of frustration and alienation. Many families felt they were not treated as individuals with unique needs, but as just another number in the healthcare system.

Becomes impersonal, and it becomes I guess… Yeah, like a business, where they push people through (Family 9).

The ability to provide culturally safe care is not simply the lack of negative feelings but the presence of knowledge and a true connection between the healthcare provider and the family receiving care. On the other hand, the families also highlighted that healthcare workers often made judgements based on stereotypes or assumptions, rather than taking the time to understand their specific circumstances or cultural context. This judgmental attitude further alienated Aboriginal families, creating a barrier to effective communication and care.

It would be nice if they [paediatricians] were a bit more aware of you know cultural backgrounds (Family 5).

These findings highlight the pressing need for better cultural safety training among healthcare staff, and an ongoing commitment to learning and understanding the complexities of Aboriginal culture, history, experiences and how their own understanding of culture facilitates safe care.

[Healthcare workers] need to do some cultural awareness courses…it opens up to our culture (Family 1).

My kids, had to have a Hepatitis A injection just because they were Aboriginal (Family 6).

And that’s probably due to like less staff and those sorts of things where people don’t… That you’ve got too much to do in your allocated time, so you’ve got to do and see so many different people that are having babies, but everybody needs their own specific amount of attention, but you don’t have that amount of time to give it to everybody. So… (Family 1).

### What Worked Well

This theme highlights positive aspects of healthcare services as experienced by the Aboriginal families in this study, emphasising areas where culturally responsive care made a significant difference. Three perspectives emerged: the critical role of Aboriginal health workers in supporting Aboriginal families, the growing awareness and understanding of Aboriginal cultures among some healthcare staff, and instances where healthcare providers demonstrated a clear understanding of the families’ health needs.

### Aboriginal Roles Supporting Aboriginal Families

Most families (*n* = 6) in the study highlighted the importance of having established Aboriginal roles within the healthcare system to support Aboriginal families.

Aboriginal assistance was not there. Yeah. It was nothing. Because, a lot of people weren’t aware of the Indigenous programmes, if they were out there (Family 1).

There was no service that they offered to me when I was pregnant (Family 7).

More specific Aboriginal roles, supporting Aboriginal families (Family 4).

They saw these roles as crucial for bridging the cultural gap between Aboriginal patients and the predominantly Western healthcare model. Aboriginal health workers bring cultural understanding and sensitivity to the care process and act as advocates and mediators, helping families navigate complex healthcare systems that may otherwise feel intimidating or inaccessible.

### Increased Awareness of Aboriginal Cultures and Understood Families’ Health Needs

Four families (*n* = 4) in the study expressed the view that healthcare services have improved in recent years, particularly in terms of increased awareness of Aboriginal cultures among healthcare staff.

The cultural awareness and the understanding of Indigenous is definitely out there now. And I just hope people take that onboard and utilise it [training]. Because that what it’s there for (Family 1).

These families noted that, compared to past experiences, there is now a greater recognition of the importance of cultural sensitivity, with more healthcare workers demonstrating respect for Aboriginal traditions, values, and ways of communicating.

But in today’s times, even with my daughter, and especially my granddaughter. Yeah. I think that they are very respectful (Family 3).

This increased awareness made families feel more comfortable and respected when accessing healthcare services, as staff were more engaged with them in a culturally appropriate manner. The families highlighted that healthcare workers were trying to understand their cultural backgrounds, listen to their concerns, and approach care in ways that honoured their values. For instance, staff were more willing to engage in open conversations about the cultural significance of certain health practices and seek input from Aboriginal patients when making decisions about their care.

In the hospital they were given all of that information (Family 1).

When, with the ear problem, it was said that Aboriginal children have different sinus passages, so they’re prevalent to ear infections and things like that. I felt that was helpful (Family 6).

For many families, culturally unsafe practices created barriers to care, highlighting the need for systemic changes. This depersonalised approach eroded trust and made it difficult for families to feel comfortable and understood within mainstream healthcare services. However, despite this lack of trust, positive experiences also emerged, where healthcare providers successfully incorporated culturally respectful approaches, leading to improved trust and outcomes for Aboriginal families. But to achieve true equity in healthcare, services could consider and accommodate the diverse needs of all individuals, including those who may be disadvantaged by the increasing reliance on online systems. This growing cultural awareness among healthcare workers created a more inclusive and supportive environment, helping Aboriginal families feel heard and valued as individuals rather than simply patients. Overall, this theme highlighted where mainstream health falls short in meeting the needs of Aboriginal families despite evidence of improvement in some areas.

### Care Is Lost When You Get Jumbled Around

This theme highlights the detrimental impact of inconsistent care of Aboriginal families within the healthcare system. For Aboriginal families, the absence of consistent, familiar healthcare providers not only diminished the quality of care but also created feelings of frustration, anxiety, and mistrust, making it more difficult for them to fully engage with the healthcare system.

Lots of things were missed. Like now and he’s been diagnosed with autism, and I think why was this not picked up on earlier (Family 6).

### A Familiar Face

Most families (*n* = 7) in the study stressed the importance of having familiar faces throughout their healthcare journey, emphasising that consistency in healthcare workers plays a vital role in their overall experience and well-being.

It would have been nice to have someone that was a regular there that knew every time you went there what happened the month before. I always had someone different. So that was always difficult (Family 2).

For Aboriginal families, seeing the same healthcare worker across multiple appointments builds trust, strengthens relationships, and creates a sense of safety. A familiar healthcare worker not only understands the family’s medical history but also appreciates their cultural background, values, and specific health concerns, which can be overlooked when care is fragmented.

I felt that the CaFHS service to me was not acceptable at the time. Because we had so many people every time? Like, it was a different nurse every time we came. There was just no-one there that knew you from before. To have that familiar face or someone that had known you and your birth and all, that would have been a lot better for me (Family 3).

Having a consistent healthcare provider means that families do not have to repeatedly explain their needs, preferences, or cultural context, reducing the emotional strain often associated with interacting with different professionals who may not fully understand their situation.

I guess continue on with that person, sort of thing, so you don’t have to chop and change, and tell your story, because you could have a real shit day and someone could write something in your file, that you’re a bit disruptive or something like that, when you’re having a bad day or having a bad everything (Family 9).

This continuity allows for more personalised care, as familiar healthcare workers notice subtle changes in the family’s health or circumstances, which might go unnoticed by someone new and can foster open communication, as Aboriginal families may feel more comfortable discussing sensitive issues or concerns with someone they know and trust.

That lady [registered nurse] used to visit with the Aboriginal worker, so she got to know you beforehand (Family 6).

### Judgement

Some families (*n* = 4) in the study shared experiences of feeling discriminated against or judged within the healthcare services they accessed, highlighting a significant barrier to receiving equitable and culturally safe care.

I guess, if something negative or something odd is in there, then there’s a prejudgement from the person that’s trying to help you, in their head already before they even meet you. …they just read your file and if there’s something negative there, that worries you… And then, you don’t get the same sort of, I guess care, or the same sort of time as other people would (Family 9).

These families reported that healthcare workers often made assumptions based on stereotypes or preconceived notions about Aboriginal people.

That’s just typical…you know, because he [baby] was darker skin, I had comments like, you know, you feel like, they judge because he is Aboriginal (Family 5).

This judgmental attitude created an environment where families felt unwelcome, misunderstood, and reluctant to engage with the services they could benefit from.

### Systems within Systems

This theme highlights the challenges Aboriginal families (*n* = 8) face when trying to navigate the complex, layered structure of the South Australian mainstream healthcare system.

I just felt like I didn’t know where to go to get help (Family 6).

Many families described feeling overwhelmed by the sheer number of services and providers available, and the lack of clear guidance on how to access the right care for their specific needs. They said that this intricate system, which often includes multiple appointments with different specialists, clinics, and services, felt like a maze, especially for families unfamiliar with how these systems operate.

Yeah, it was a lot of different locations that you went to, whether it be from your GP to the actual Hospital, to, uh, the parenting course which was somewhere else. And we also used, uh, another service where our daughter had, uh, an allergic reaction to a certain type of chemical. So, we went to another meeting with a few people to try and help deal with those problems and things like that. And that also was at a different spot again, so everything is sort of jotted around Adelaide (Family 9).

For Aboriginal families, the difficulties in navigating this system are compounded by the absence of culturally appropriate support or assistance in understanding how the services connect and how they can benefit them.

I think we had to organise it ourselves, because there’s no sort of set plan when you have a child. Just scattered around. I had quite a helpful partner that was good at researching all those sorts of things, so she really looked out and looked after all that sort of side (Family 3).

### You Lose Care Being Jumbled Around

This theme reflects the frustration and emotional toll experienced by Aboriginal families as they navigated a healthcare system characterised by fragmented and inconsistent care. A common complaint from the families in the study (*n* = 8) was the need to repeatedly share their personal health stories with multiple healthcare workers who seemed unfamiliar with their medical history, cultural background, or even basic details about their children.

So, it was a little bit hard to relate to anybody because it’s sort of just meeting a random stranger, and then they’re telling you I guess intimate details, how to do things and stuff like that. So, it’s a bit impersonal (Family 9).

Families expressed frustration over the lack of continuity, feeling that healthcare workers did not know them or their babies. This lack of familiarity often left them feeling like their concerns were not being fully understood or appropriately addressed, as each new healthcare provider was starting from scratch, without the context of previous interactions.

Because they don’t know where you’re at with the baby or where we’re at with the pregnancy and stuff like that. Yeah. It was frustrating (Family 8).

For parents, especially those navigating complex or ongoing health issues, it was particularly distressing that staff did not recognise the nuances of their baby’s care history. This not only led to a loss of trust in the system but also made them feel like they were just another number, rather than individuals with unique health needs.

You’re just sort of another number (Family 2).

Someone that doesn’t seem to care or have… Or be too busy. Sometimes they’re often too busy. Overworked and things like that. So, they don’t really have time to check in. They ask if everything’s okay, but people instinctively say yes. Yeah, it probably would have been better, because when you do tell your story, you don’t reveal all your information all the way. Particularly, I don’t let people in and all that sort of stuff. And I think they asked if I was all right and all that sort of stuff, but I would have just said yes (Family 1).

The challenge of sharing intimate health details with different providers amplified feelings of vulnerability and discomfort. Many families felt uneasy disclosing personal health histories to new healthcare workers, particularly when these interactions felt rushed or impersonal.

But I guess if it was the same midwife from five months before the pregnancy, up to the next couple of years, sort of coming over and talking, and someone that you’d known for I guess… Yeah, six months to a year, and seen them regularly, it might be easier to say if you have issues…And then, that sort of goes on with you, instead of someone actually knowing you and then realising, oh yeah, if I had have been in the same situation I would’ve been upset as well. So, I guess you lose care, being jumbled around (Family 5).

This fragmentation in care ultimately undermined the overall quality of healthcare the families received. The lack of continuity, compounded by the emotional exhaustion of repeatedly explaining their situations, resulted in a sense that important details were being missed or misunderstood.

It all just sort of seemed a bit, yeah, disjointed I guess, with no sort of layouts. It was, oh yeah, come and have a scan later on, or I guess it sort of seemed a bit more, I guess businessy, where you’re more of a customer than a… Someone that needs to be looked after (Family 8).

This experience of being *jumbled around* contributed to a perception of inadequate care, leaving families feeling unsupported, unseen, and disconnected from the healthcare system.

You know, I had some issues, diabetes and I had some low blood pressure issues, so I was doing painting and all that sort of stuff. So, one month you’d go and see one person and you tell them about that but next time, you had to repeat it all over again. And I never got a result, no-one ever resolved it (Family 2).

The findings of this theme underscore the critical need for more consistent, relationship-based care models that prioritise ongoing, familiar connections between healthcare providers and Aboriginal families to ensure that both medical and cultural needs are fully addressed. The absence of a consistent caregiver meant that families were often explaining their most private concerns to someone who had no prior relationship with them, making it difficult to build trust or feel confident that their needs would be met with care and understanding and deepened feelings of disconnection from the healthcare process.

### In and Out for Check-Ups, Scans, and Things—No One Asked If I Needed Help

This theme describes the sense of detachment and lack of support that some of the Aboriginal families (*n* = 5) experienced when engaging with the healthcare system for routine tests, check-ups, and scans. Families frequently described their encounters with healthcare services as hurried and impersonal, with appointments that felt like a quick, transactional process rather than a meaningful interaction focused on their overall well-being. These in-and-out visits often centred around medical procedures or tests and left little room for communication, guidance, or emotional support.

We had a series of, uh, midwives’ and doctors’ appointments, um, scans and all that, that sort of stuff (Family 8).

I was just in and out (Family 2).

And just recently when my granddaughter had a child. I was thinking, why is she having all these tests? (Family 1).

A recurring sentiment among families was the lack of opportunity to ask questions or seek clarification, as appointments were rushed, and healthcare workers appeared more focused on completing the required tests than ensuring the patient felt cared for. This assembly-line approach to care left families feeling isolated and unsure, with little to no opportunity for healthcare workers to inquire about their broader needs. Whether it was a routine scan or an important check-up, families reported that no one asked if they needed further assistance or support—whether related to their physical health, emotional well-being, or cultural needs.

When we moved to here, I didn’t have that support group being an older mum was just, I felt secluded (Family 2).

I just don’t think people look at, listen enough to the parent... And to be able to explore what’s [services] actually relevant or not (Family 2).

For many Aboriginal families, this lack of engagement meant that their broader health concerns, personal challenges, or cultural preferences were often overlooked.

I would’ve liked maybe some more home help. Because I didn’t seek it at the CaFHS… Maybe would’ve I liked my, someone to come round and just... Help you out. Like I said, because I had struggled with the feeding and the, you know, just a few little things. And the sleeping, and just to maybe have someone just to reassure you that everything was going okay, that was all (Family 2).

The parents just go to doctors and get called out. They actually need some education (Family 5).

There needs to be more parental sessions, more focused on putting support in place with parents who are really struggling (Family 5).

The absence of a holistic, patient-centred approach in these interactions left families feeling like their experiences and health care were fragmented and incomplete. Furthermore, the failure to ask if additional help or resources were needed—whether it be guidance on managing their health, navigating the healthcare system, or accessing culturally appropriate services—reinforced the feeling that they were not truly being listened to or cared for.

But some families felt heard and received the help they needed:

You get some fantastic people and some people that don’t want to be there, or some people that have issues or things like that. So, there’s always a good and a bad (Family 9).

I was happy because I didn’t have any complications (Family 3).

And then when she went home, she had home visits from the Indigenous health worker (Family 1).

They actually offer transport for free [to service used] (Family 5).

Some of the midwives they had in the hospital are really good, was really helpful, with fitting us in and stuff like that (Family 8).

And they came to our house a couple of times and checked up on us, and I think that was quite a good thing (Family 9).

The CaFHS nurses as well, um, went more into detail with breastfeeding, and the nutrition side of things, which was good (Family 8).

We found a couple of groups, For Mum’s Groups, and things like that. They had Father’s Groups as well, and post-traumatic stress disorder… post-natal depression, that’s the one (Family 9).

One family commented on living away from the metropolitan area in a smaller area:

And then the hospital up there, I think being a smaller community we knew more people. And so, you felt a lot more relaxed and supported (Family 2).

Families in the study (*n* = 6) emphasised the crucial role that family support played in their healthcare journey.

I was lucky that I had family that could help me. Because if you are a mum in rural and you didn’t have family to help you, that would have been a bit tricky (Family 3).

Participants noted that having family members involved provided emotional strength, practical assistance, and a sense of cultural grounding, which was especially important when navigating the healthcare system. Family presence was seen as helping to alleviate feelings of isolation and reinforced the collective nature of care in Aboriginal culture.

I had, um, my mum and my, um, mother-in-law which helped a lot as well, which was really good. And I didn’t get sleep, and they would come down and help so that I can have a rest (Family 7).

Overall, this theme demonstrated a desire for greater Aboriginal involvement in health care, encouraging greater involvement of family members and a less complex and hurried system. However, the lack of clear pathways or a central point of contact to help guide Aboriginal families through referrals, appointments, and follow-up care means that families struggle, resulting in missed appointments, delays and are shuffled between services without a clear understanding of who is responsible for their care. A recurring issue expressed by families was the lack of continuity of care, where recipients of care are frequently passed between different healthcare providers or services, leading to fragmented care.

## Discussion

A key theme that emerged from this study is the need to bridge the gap between the unique care needs of Aboriginal families and Western models of care in mainstream services. One way to bring culture to the centre of health care services was to establish specific Aboriginal health roles in the mainstream health care systems and service delivery. The presence of Aboriginal health workers (AHWs) can make women and families feel more comfortable and supported, as these workers understands their cultural values, communication styles, and the importance of kinship and community in health decision-making [5]. Aboriginal health professionals are uniquely positioned to provide culturally safe care by offering empathy and understanding that resonates with the lived experiences of their communities. This not only enhances the quality of care but also builds trust, which is vital for encouraging Aboriginal families to engage more consistently with healthcare services [12, 13]. Families in this study felt that AHWs played a key role in ensuring their voices were heard and their health needs were taken seriously by the broader healthcare team. By having a shared cultural background, AHWs could improve communication in regard to concerns and preferences of Aboriginal families to non-Indigenous healthcare providers, ensuring that care was more aligned with the families’ needs and values [1, 7]. This support can help to mitigate feelings of isolation or being misunderstood, which many Aboriginal families encounter when navigating mainstream healthcare [14]. The presence of Aboriginal roles within healthcare is seen as a vital factor in improving health outcomes for Aboriginal families and ensuring the system is responsive and inclusive [17]. However, there are few Aboriginal health professionals to fill this role hence non-Aboriginal healthcare professionals must adopt an allyship with Aboriginal people to ensure this support is available [20]. Families in this study saw an encouraging shift toward a more equitable healthcare system, where cultural differences are acknowledged and respected. While challenges remain, the families’ positive experiences indicate progress and highlight the importance of continued efforts to enhance cultural inclusion in healthcare. Another South Australian study [1, 7] found that culture at the centre of care could lead to better health outcomes and a stronger sense of trust between Aboriginal families and healthcare providers. This increased awareness also reflects a broader shift in the healthcare system’s approach to inclusivity, moving toward a more culturally safe model of care that seeks to address the unique needs and preferences of Aboriginal communities.

For Aboriginal families, continuity of care can also help reduce feelings of being judged or misunderstood, which are common experiences when engaging with multiple, unfamiliar healthcare providers [12]. A familiar healthcare worker provides a sense of stability and assurance, reinforcing that their care is not only clinically safe but also culturally safe. This consistency is particularly important for Aboriginal families, where relationships and trust are key components of well-being, making the presence of familiar faces a critical factor in delivering effective and culturally safe healthcare [9].

Families in this study experienced a sense of judgement by some healthcare workers. Discrimination can manifest in various ways, including dismissive or indifferent behaviour from healthcare staff, as well as a lack of empathy and a reluctance to listen to the specific needs and concerns of the families [15]. For instance, some healthcare providers may attribute someone’s health issues to negative stereotypes, such as assumptions about lifestyle or adherence to treatment, rather than taking the time to explore the actual context of their lives. This not only undermined peoples’ confidence in the care they were receiving but also reinforced feelings of being “othered” or marginalised within a system that should be supportive [16]. Experiencing judgement or discrimination may contribute to women and families feeling less inclined to seek care in the future, fearing further mistreatment or misunderstanding [1]. The emotional toll of being judged or discriminated against, particularly when dealing with sensitive health matters, can deepen mistrust of the healthcare system. This mistrust can lead to delayed or avoided care, which exacerbates existing health disparities among Aboriginal populations [15]. This emphasises the urgent need for healthcare providers to undergo more comprehensive cultural safety training and for healthcare services to actively work toward creating more inclusive, respectful, and non-judgmental environments.

Without addressing these issues, Aboriginal families will continue to experience inequitable care, which can have lasting negative effects on their health and well-being [1, 7]. Similarly, the families’ experience of systems within systems, the healthcare system’s bureaucratic nature can further alienate families, as they encounter multiple forms of administration and varying levels of service delivery without adequate explanation or support [1, 7]. Aboriginal families in this study reported that this made them feel disconnected from the care process and that their individual needs were being overlooked. This lack of support also highlights a broader systemic issue, where families are not provided with the resources or information necessary to effectively engage with healthcare services. The disconnect between services, the complicated referral systems, and the lack of personal, culturally safe assistance means that Aboriginal families often fall through the cracks of the system, further deepening existing health inequities [1, 7].

Addressing this issue requires not only simplifying the healthcare navigation process but also offering personalised, culturally safe guidance to help Aboriginal families confidently access the care they need. There is a need to slow down healthcare interactions and focusing on more than just the technical aspects of care. Families need healthcare providers to take the time to check in, ask meaningful questions, and ensure they are supported throughout their healthcare journey—not just when tests or procedures are required. This approach is especially important for Aboriginal families, who may already face barriers to accessing culturally safe care. By providing more personalised, attentive, and culturally informed care, healthcare workers can better ensure that Aboriginal families feel valued, supported, and empowered to engage with the mainstream healthcare system.

## Limitations

This study has several limitations that should be considered when interpreting the findings. Firstly, the sample size was relatively small, with only nine families participating, which may limit the generalisability of the results. The qualitative design, while valuable for in-depth insights, also means that the findings reflect the specific experiences and perceptions of this participant group and may not capture the diversity of experiences among all Aboriginal families. Additionally, the study focused on mainstream healthcare services in South Australia, so findings may not be applicable to other regions where healthcare systems and community contexts vary. Additionally, Aboriginal and Torres Strait Islander community and personal needs vary enormously across Australia; hence, these findings cannot be applied to Aboriginal people and communities in general. Lastly, the use of yarning methods, while culturally appropriate and effective in fostering open dialogue, may introduce subjectivity, as the data interpretation relies on the researchers’ understanding of participants’ narratives.

Despite these limitations, this study offers several strengths. The use of yarning as a culturally respectful and effective qualitative method allowed Aboriginal participants to share their experiences in a comfortable and supportive setting, promoting trust and openness in discussing sensitive healthcare topics. The focus on continuity of care is particularly valuable, as it addresses a gap in understanding the barriers and facilitators to positive healthcare experiences for Aboriginal women and their infants. By identifying specific themes and strategies that promote culturally safe and coordinated care, this research provides actionable insights for improving service delivery in mainstream healthcare settings. The findings highlight the importance of cultural inclusion and continuity in healthcare, offering practical recommendations that can contribute to reducing health disparities and enhancing trust between Aboriginal communities and mainstream healthcare providers.

## Conclusion

This study highlights the critical role of culturally safe, continuous, and well-coordinated care in improving health outcomes for Aboriginal women and their infants. The findings reveal that Aboriginal families often face challenges within mainstream healthcare services, including a lack of continuity, inadequate communication, and limited cultural understanding, which can lead to fear, anxiety, and lower service attendance. By centring culture in healthcare practices, reducing fragmentation in care delivery, and fostering respectful, consistent relationships with healthcare providers, services can better meet the needs of Aboriginal families. The insights gained from this study provide actionable strategies for enhancing the quality and accessibility of maternal and child healthcare for Aboriginal communities in South Australia. Implementing these culturally appropriate practices has the potential to bridge healthcare gaps, reduce disparities, and build trust between Aboriginal families and mainstream healthcare services, contributing to healthier outcomes across generations.

## Data Availability

The datasets used and/or analysed during the current study are available from the corresponding author on reasonable request.

## Declarations

### Terminology

In Australia, care must be taken to ensure appropriate terminology when referring to Aboriginal and Torres Strait Islander people and communities. This study has followed Aboriginal and Torres Strait Islander guide to terminology endorsed by the Public Health Association of Australia. Please note that in this study the term Aboriginal is used; however, we acknowledge that there is great diversity in the Aboriginal population (Public Health Association Australia, 2023).
